# Transcriptome-scale homoeolog-specific transcript assemblies of bread wheat

**DOI:** 10.1186/1471-2164-13-492

**Published:** 2012-09-19

**Authors:** Andreas W Schreiber, Matthew J Hayden, Kerrie L Forrest, Stephan L Kong, Peter Langridge, Ute Baumann

**Affiliations:** 1 Australian Centre for Plant Functional Genomics, Univ. of Adelaide, PMB 1 Glen Osmond, SA 5064, Australia; 2 Department of Primary Industries Victoria, Victorian AgriBiosciences Centre, La Trobe Research and Development Park, Bundoora, VIC 3083, Australia; 3 ACRF South Australian Cancer Genome Facility, SA Pathology, Frome Road, Adelaide, SA 5000, Australia

**Keywords:** Wheat transcriptome, Wheat genes, Sequence assembly, Cloud computing

## Abstract

**Background:**

Bread wheat is one of the world’s most important food crops and considerable efforts have been made to develop genomic resources for this species. This includes an on-going project by the International Wheat Genome Sequencing Consortium to assemble its large and complex genome, which is hexaploid and contains three closely related ‘homoeologous’ copies for each chromosome. This multi-national effort avoids the complications polyploidy entails for correct assembly of the genome by sequencing flow-sorted chromosome arms one at a time. Here we report on an alternate approach, a direct homoeolog-specific assembly of the expressed portion of the genome, the transcriptome.

**Results:**

After assessment of the ability of various assemblers to generate homoeolog-specific assemblies, we employed a two-stage assembly process to produce a high-quality assembly of the transcriptome of hexaploid wheat from Roche-454 and Illumina GAII_x_ paired-end sequence reads. The assembly process made use of a rapid partitioning of expressed sequences into homoeologous clusters, followed by a parallel high-fidelity assembly of each cluster on a 1150-processor compute cloud. We assessed assembly quality through comparison to known wheat gene sequences and found that in ca. 98.5% of cases the assembly was sufficiently accurate for homoeologous triplets to be cleanly separated into either two or three separate contigs. Comparison to publicly available transcript collections suggests that the assembly covers ~75-80% of the complete transcriptome.

**Conclusions:**

This work therefore describes the first homoeolog-specific sequence assembly of the wheat transcriptome and provides a reference transcriptome for future wheat research. Furthermore, our assembly methodology is transferable to other polyploid organisms.

## Background

Rapid increases in sequence output and read length of next generation sequencing instruments, accompanied by reduced error rates, are revolutionising molecular biology [1,2]. As of May 2011, sequencing projects of 1543 prokaryotic and 39 eukaryotic organisms, including five plants, are listed by NCBI as being ‘completed’ [3]. Indeed, as opposed to only a few years ago, the technological progress has been so great that nowadays the main difficulty has shifted from sequence acquisition to sequence assembly [4].

The difficulty of sequence assembly is the most significant factor explaining the relatively small number of plant species with finished genome sequences. Plant genomes tend to be large and highly repetitive, they have a propensity to contain large gene families and are frequently polyploid. For example, the wheat genome is estimated to be 90% repetitive [5], it is hexaploid, and at 16 Gb it is roughly 5 times the size of the human genome. Any computational procedure for assembling such large and complex genomes must, therefore, be exceedingly efficient with both time and memory resources, but at the same time must be highly accurate to avoid mis-assembly of closely related sequences. For this reason, the sequencing of bread wheat (cultivar Chinese Spring) by the International Wheat Genome Sequencing Consortium [6,7] is being carried out on individual flow-sorted chromosomes [8,9] to (at least) avoid the difficulty of simultaneous sequence assembly of closely related homoeologs. Nevertheless, the significant challenges associated with assembling repetitive genomes [10,11] make it likely that the availability of a finished, reliable, wheat genome sequence is still a number of years down the track.

Wheat is one of the world’s most important food sources with a total world production of ~683 × 10^6^ metric tons [12]. It ranks with rice (689 × 10^6^ tons) and maize (827 × 10^6^ tons) among the world’s most cultivated crop plants. Worldwide, significant efforts are underway to use modern biotechnology to assist wheat breeding programs to increase crop yield, nutritional content, salinity and drought tolerance, as well as biotic tolerance [13]. While knowledge of the full genome sequence of wheat is undeniably highly desirable for wheat improvement, for many purposes knowledge of the expressed portion of the genome, i.e. the transcriptome, is sufficient. For example, wheat transcriptome sequencing can be used to identify candidate genes for trait expression and to develop SNP markers for tracking favourable alleles in breeding programs. Knowledge of the transcriptome also greatly aids the design of microarrays and the interpretation of RNA-Seq experiments [14]. In one sense, assembly of the transcriptome is more straightforward than assembly of the genome in that one largely does not need to worry about the repetitive sequences that plague the latter. Nevertheless, direct sequencing and assembly of the transcriptome of bread wheat is also not without challenges due to the presence of up to three highly similar homoeologs per locus.

In this contribution, we describe our work towards the sequencing and subsequent homoeolog-specific assembly of the wheat transcriptome and show, through comparison with existing sequence resources, that the resultant assembly goes a long way towards producing a comprehensive compendium of the gene sequences of bread wheat. As far as we are aware, a homoeolog-specific as opposed to homoeolog-blind assembly (see, e.g., [15]) of a polyploid transcriptome has not been performed before. After testing various assembly algorithms for their ability to produce homoeolog-differentiated assemblies, we decided on an assembly using a two-stage approach. First, a rough assembly was produced using the Velvet/Oases assembler [16,17]. This assembly was found to be mostly insensitive to the slight sequence differences between homoeologs, previously estimated to be about 1 SNP per 145 bases [18]. It is convenient to think of the Velvet/Oases assembly as consisting, for the most part, of sequences with simultaneous contributions from the A, B and D genomes and, possibly, recent gene duplications. This assembly was used only to group reads into convenient clusters. In the second stage, reads in each cluster were re-assembled separately, using the high-precision assembler MIRA [19-21]. This assembler was found to be sufficiently sensitive to differentiate homoeologs in most cases. The two-stage approach was adopted because, given current computing (particularly memory) constraints, the direct assembly of a complex, polyploid, eukaryotic transcriptome using MIRA alone is not yet feasible. Because the assembly of each cluster could be done independently, the second step was implemented in a highly parallel fashion on a compute cloud.

## Results

### Sequencing of the wheat transcriptome

Wheat mRNA from a single cultivar, the elite variety “Kukri” [22,23], was sequenced using: a) short-read Illumina GAIIx technology for sequencing depth, and b) long-read Roche GSFLX Titanium technology for homoeolog-sensitivity. RNA was extracted from root and shoot tissue from seedlings ranging from 8–12 days after germination, as well as florets collected at various stages from pre-meiosis to just prior to anthesis. Collection from multiple tissues and developmental stages was essential in order to obtain a reasonably comprehensive representation of the complete transcriptome. The RNA was normalized [24] in order to reduce the dominance of abundantly expressed genes.

After quality checks, trimming of adapters and size selection using custom scripts (see Methods) 14,563,748 Illumina GAIIx reads (6,913,826 read pairs, insert size ~250-300 bases, and 736,096 single reads; mean sequence length 107.8 bases) and 1,495,941 GS FLX sequences (mean sequence length 363.2 bases), i.e. 16,059,689 reads in total, were used as input to the sequencing assembly pipeline.

### Assembly algorithm performance testing

**Figure 1:**
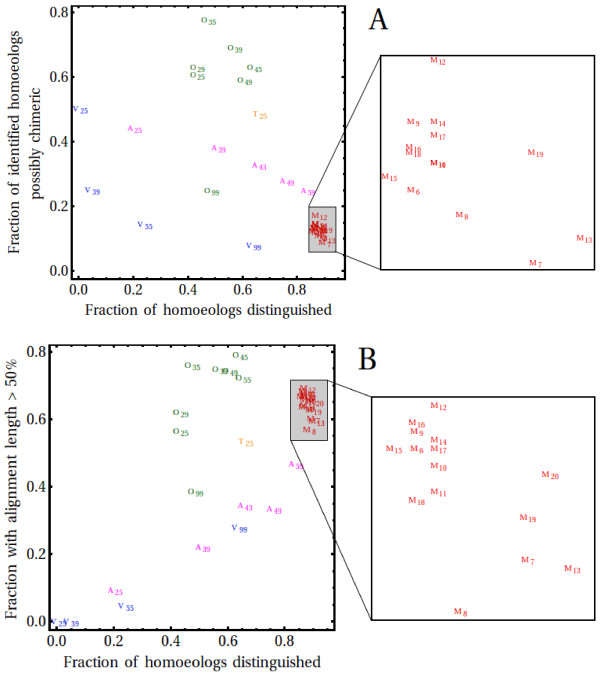
**Performance of various assembly algorithms.** Assembled sequences were assessed by comparison to a reference set of 65 homoeologous triplets (A: ABySS, V: Velvet, O: Velvet/Oases, M: MIRA). Results for the transcript assembler Trinity (T), which has become available more recently, are also shown as a comparison. Subscripts on A, V, O and T indicate k-mer size; subscripts on M indicate assembly parameters as listed in Additional file 1: Table S1. Panel **A** shows the fraction of homoeologs identified (%ID >98%) vs. the fraction of contigs with evidence of chimeric assembly (for details, see Methods). A perfect assembly would appear near the bottom right hand corner of the plot. Note the high number of chimeric assemblies, i.e. lack of homoeolog-specificity, exhibited by the de-Bruijn graph-based Oases and Trinity assemblers. The larger k-mer sizes approach the average length of the Illumina reads, thereby decreasing the coverage per contig. Panel **B** shows the fraction of homoeologs identified (%ID >98%) plotted against the fraction of contigs with an alignment length larger than 50% of the relevant homoeolog length (see Methods), giving an indication of the fraction of sequence covered by individual contigs. In this panel, a perfect assembly would appear towards the top right hand corner of the plot. Note that the Velvet/Oases assemblies tend to produce the longest contigs, but at the expense of homoeolog-specificity (Panel **A**).

We investigated the suitability of various assembly algorithms by comparison to 65 validated bread wheat (cv. Chinese Spring) homoeologous sequence triplets (the “OM” set; see Methods), obtained from [18] and [25]. Reads that bore some similarity to sequences in the OM dataset were extracted from the Illumina and GS FLX reads and assembled, using various parameters, with ABySS ([26], Version 1.2.6), Velvet ([16], Version 1.0.18), Velvet/Oases (Version 0.1.18) as well as MIRA ([19,20] Version 3.2.1; [19,20]). Assembled contigs were subsequently compared to the OM homoeologs, as described in Methods, and evaluated according to criteria such as evidence of chimeric sembly of homoeologs, sequence length, total number of homoeologs assembled, etc. As can be seen in Figure 1A, genome assemblers such as Velvet and ABySS tend to produce a significant number of chimeric assemblies of homoeologous copies of each gene, thereby reducing the total number of homoeologs in the assembly. The chance of chimeric assembly decreased as the k-mer length associated with the underlying de-Bruijn graph was increased, as one might expect. Usage of the transcript assembler Velvet/Oases significantly increased the rate of chimeric assembly to around 60-80%. While this was undesirable, the lack of homoeolog-specificity allowed Velvet/Oases to produce significantly longer contigs than Velvet and ABySS (Figure 1B). We found that MIRA, which is not a de-Bruijn graph-based assembler, exhibited good homoeolog-specificity (Figure 1A) over a wide range of assembly parameters (Additional file 1: Table S1), without significantly compromising contig length (Figure 1B), but at the expense of prohibitively increased memory and CPU-time requirements. For comparison, Figure 1 also shows results obtained with Trinity, another short-read de-Bruijn graph based assembler developed more recently ([27], Version 5-19-2011). Homoelog-specificity (Figure 1A) obtained with this assembler is somewhat better than that obtained with Velvet/Oases, but considerably worse than that obtained with MIRA, with evidence for chimeric assemblies in around 50% of cases. Coverage is reduced somewhat (Figure 1B) compared to Velvet/Oases, presumably reflecting the greater homoelog-specificity.

### Sequence assembly

In view of the results shown in Figure 1, a two-step assembly procedure using Velvet/Oases and MIRA was employed. Initially, a total of 16,059,689 Illumina and GS FLX reads were assembled with Velvet/Oases as described in Methods. This assembly resulted in a total of 69,975 contigs, with an average length of 840 bases. Using the number of genes in rice (~41,000; [28]) as a guide, and taking the polyploidy of wheat into account, it is to be expected that the total number of wheat genes significantly exceeds this number of contigs. This supports the conclusion reached during assembly testing against the OM set that the Velvet/Oases assembler is largely homoeolog insensitive. Definitive conclusions, however, are difficult to draw because not all genes would have been expressed in the samples that were sequenced (decreasing the expected number of contigs), while alternate splice-forms would tend to separate in the assembly (increasing the expected number of contigs).

**Figure 2:**
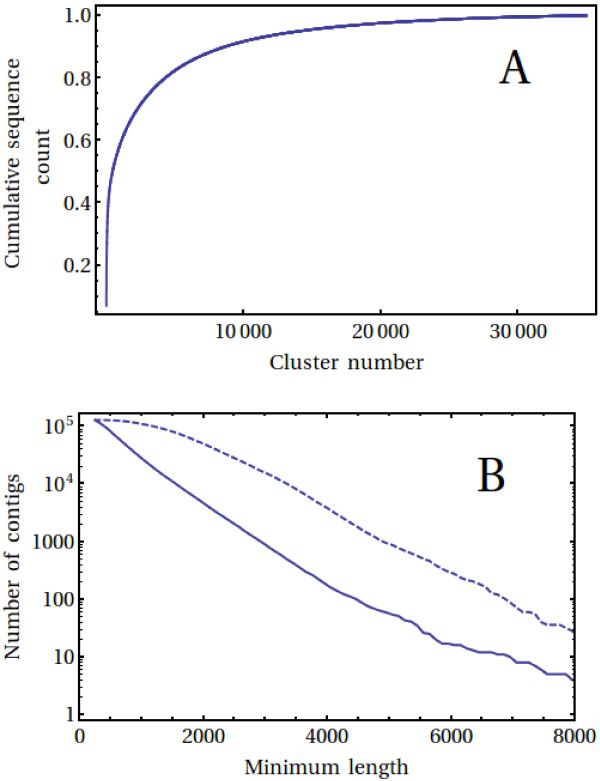
**Assembly statistics.** Panel **A** displays the cumulative count of the number of reads in each cluster, while Panel **B** shows the length distribution of the contigs. Note that 95% of reads were contained in the largest 14,000 clusters (Panel **A**) and almost 28,000 wheat contigs (solid line, Panel **B**) are longer than 1,000 bases. For comparison, the length distribution of rice cDNA sequences is also shown (dashed line, Panel **B**).

In order to produce clean homoeolog-specific contigs, the Velvet/Oases contigs were subsequently used purely to cluster the initial reads, as described in Methods, and then discarded. Importantly, as we wanted to avoid individual (possibly mis-assembled) homoeologs ending up in separate clusters at this early stage, we also included a lenient clustering of the Velvet/Oases contigs in this step. 78% of reads could be clustered in this way, with the largest cluster containing 24,806 GS FLX and 1,066,593 Illumina reads. The largest 14,000 clusters contained just over 95% of the total number of reads (Figure 2A). In each cluster, the number of GS FLX reads amounted to, with large fluctuations, 15-20% of the total number of reads in that cluster.

Next, each read cluster was assembled separately using MIRA implemented on a compute cloud, as described in Methods. After filtering for quality (average base error probability <10^-4^) and contig length (>250 bases), 128,628 contigs were left in the assembly. Their cumulative size distribution is shown in Figure 2B, with 27,958 contigs larger than one thousand bases and the longest contig being 11,572 bases. For comparison, the cumulative size distribution of rice cDNAs is also shown (rice cDNA sequences were obtained from [28], with those rice sequences starting with ATG discarded as they did not contain the 5’ UTR region; the total number of sequences was normalized to that of the wheat assembly for comparison). As can be seen, while the cumulative distributions are of similar shape, in general the assembled wheat contigs are shorter than the rice cDNAs. While longer contigs could presumably be obtained by reducing assembly stringency, this would in general reduce contig accuracy and, in particular, homoeolog specificity.

Sequences were annotated through comparison to rice cDNAs, and assigned to map locations when available, as described in Methods. In this way, almost 78% of sequences could be annotated through the rice cDNAs, while 27,694 sequences (including 1,667 over 1,000 bases long) could not be identified.

### Sequence coverage

Characteristics of transcriptome coverage provided by the assembly were estimated by comparison to a set of 6,166 full length cDNA clones (“FLs”; [29,30]) as well as the Harvard Tentative Contigs (“TCs”; [31,32]) that are themselves assembled from ESTs in public databases. While it is difficult to be certain about the level of homoeolog-specificity in the TCs, the assembly algorithm that generated them (see Methods) makes it likely that they are dominated by the most abundantly expressed homoeologs. ESTs originating from homoeologs with lower expression are likely to be frequently mistaken as sequencing errors of the more abundant member of the homoeologous group.

**Figure 3:**
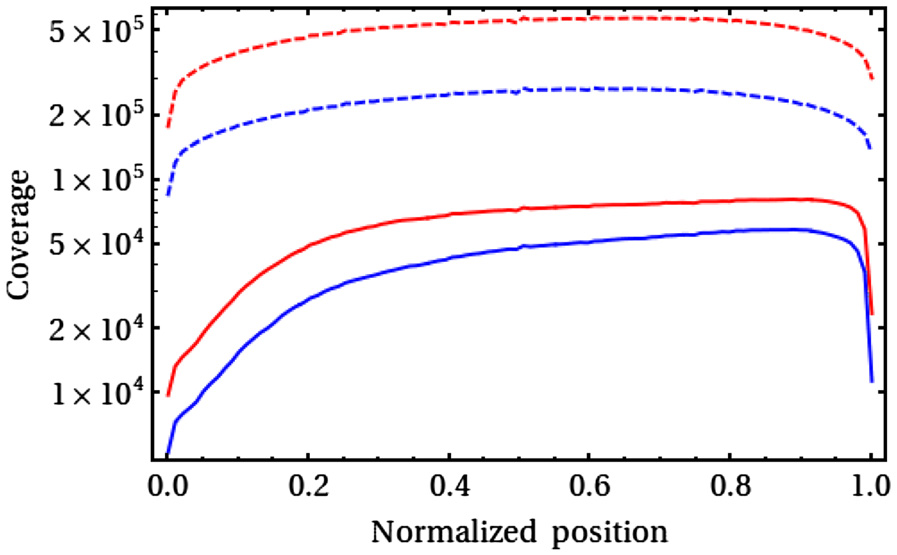
**Coverage of full length wheat cDNAs and tentative contigs by our assembled sequences.** The individual curves correspond to lower cut-offs for the %IDs required to define a match (from top to bottom, 90 and 99%, respectively; E-value <10^-50^ for all curves). Coverage of full length cDNAs [29] are shown as solid lines, coverage of the Harvard tentative contigs [32] as dashed lines. Nucleotide positions are relative to the total length of the full length cDNAs and tentative contigs, respectively.

In Figure 3, we show the average coverage along the length of the FLs and TCs as a function of the minimum%-identity demanded for a match (% identity, %ID for short, refers to the similarity of the highest scoring pair in a BlastN hit; only BlastN hits with an E-value <10^-50^ are considered). The coverage is fairly uniform, only dropping off significantly in the first 20% and in the last 5% of the length of the FLs. The distributions for the TCs are more symmetrical than for the FLs as the former’s orientation is, in general, not fixed. The average coverage of the FLs drops by a factor of around 3.0 (2.7 for the TCs) when moving from low specificity (%ID~90%) to high specificity (%ID~99%). As discussed below, we expect a %ID of 90% or less to be largely homoeolog-insensitive, while a %ID of 99% or more likely to be homoeolog-discriminating. The drop by a factor of around two or three is, therefore, consistent with the assembly being a homoeolog-discriminating assembly of a (homozygous) hexaploid.

5,034 of 6,166 (81.6%) FLs were matched by at least one assembled contig at 90% identity. This reduced to 4,110 (66.7%) at 99% identity. For 1,439 and 642 of these, respectively, the alignment extended for more than 80% of the length of the FL sequence. For the TCs, 69,187 out of 93,508 (74.0%) were matched by a least one assembled contig at 90% identity and 39,430 (42.2%) at 99% identity. We note, however, that at 99% ID the results have a strong dependence on the %ID (e.g., at 98 %ID, the numbers are 74.2% and 54.7% for the FLs and TCs, respectively). We comment on the implications of this in the Discussion.

### Homoeolog specificity

**Figure 4:**
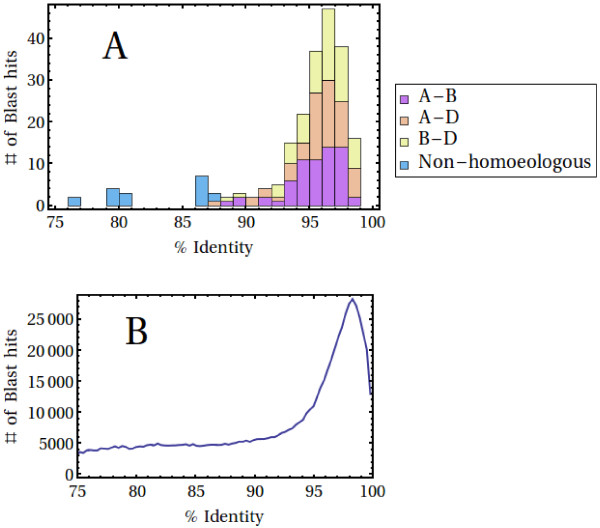
**Sequence similarity of assembled contigs compared to those of the OM sequence set.** Similarities between homoeologs from the OM set (Panel **A**) are all between 87 and 99%, while similarities between a small set of genes not classified as homoeologs in Ogihara et al. [25] and Mochida et al. [18] exhibit a somewhat smaller sequence identity. Sequence similarities between assembled contigs (Panel **B**) were quantified from an all-vs-all BlastN search (E-value < 10^-50^, alignment length >400 bp). A sharp rise at a %ID at around 93% is clearly visible. Note that a maximum of one Blast hit per sequence pair has been retained in order to produce this plot.

The quality of the final assembly was investigated by again comparing the results to the 65 validated bread wheat (cv. Chinese Spring) homoeologous sequence triplets of the OM set, as described earlier. The sequence diversity of the OM sequences is shown in Figure 4A. An all-vs-all BlastN comparison was used to quantify this diversity, with hits with an E-value better than 10^-50^ and an alignment length larger than 400 bases being retained. As can be seen, homoeologs in the OM set predominantly exhibit a %ID of between 93 and 99%.

The sequence diversity of our own assembly was quantified in the same way and is shown in Figure 4B. This plot is normalized in such a way that at most one hit per sequence pair, with the greatest sequence similarity, is retained. A sharp rise in sequence-pairs with %ID above 93% may again be observed and it is tempting to conclude, by comparing to Figure 4A, that this peak above background is largely caused by the presence of homoeologous sequences. If this is indeed the case, one would deduce from Figure 4B that the average sequence identity of homoeologs in wheat is about 97.2%, with a spread (standard deviation) of about 1.8%. This in turn corresponds to a SNP frequency per sequence of ~1.4%, which is a little higher (1 SNP/71 bases) than that reported by Mochida et al. [18]. Furthermore, under the extreme assumption that the excess above background seen in Figure 4B (approx. 83,600 Blast hits) is entirely due to homoeologous sequence triplets, one ends up with a lower bound of around 27,900 × 3 transcripts in the dataset. Because in reality one would expect the RNA sample to consist of a mixture of expressed homoeologous triplets, doublets and single sequences, this lower bound, being 68% × 3 of the gene content in rice, is not unreasonable.

Next, the assembled sequences were compared to the OM set using BlastN (E-value < 10^-100^, word size 11). Sequences were allocated to individual homoeologs, using %ID as a criterion, by iteratively identifying and removing highest quality matches, as before (see Methods). Results of this comparison are shown in Additional file 2: Table S2. On average, the %ID of the identified matches is 99.67%. The SNP frequency between varieties of bread wheat is naturally somewhat variable and dependant on cultivar and lineage, with published estimates ranging from 1 SNP/540 bases [33] to 1 SNP/335 bases [34]. It appears likely, therefore, that the 1 SNP/300 bases observed between the assembled Kukri sequences and the OM set (Chinese Spring) can largely be attributed to inter-cultivar polymorphisms.

In total, this procedure identified 186 out of a total of 195 (95.4%) possible homoeologs in the OM set. For 57 triplets all three homoeologs were identified (87.7%), for 7 triplets two out of three homoeologs were identified (10.8%) and in one case only one member of the triplet was identified.

The level of chimeric assembly between reads originating from separate homoeologs was quantified in the final assembly, as described earlier. In total, only 27 out of 186 (14.5%) assigned sequences showed some evidence of chimeric assembly.

These results are almost identical to those shown for the assemblies produced by MIRA and shown in Figure 1, which did not involve an initial Velvet/Oasis assembly. This is consistent with the interpretation that the two-stage assembly was not compromised by mis-assembly in stage one.

Finally, a set of 19 random clusters was selected from the final assembly in such a way that 3 contigs therein were mutually overlapping. These contigs were then compared to unassembled reads from a genomic wheat sequencing project of the variety Chinese Spring ([35]; see Methods). The existence of any reads that could clearly be associated with more than 1 contig was taken as evidence of chimeric assembly. In total, 11 suspect contigs were identified in this way, leading to an independent estimate of the rate of chimeric assembly of about 18%. This is in rather good agreement with the estimate provided by the comparison to the OM set described earlier.

### Comparison to other grass genomes

**Figure 5:**
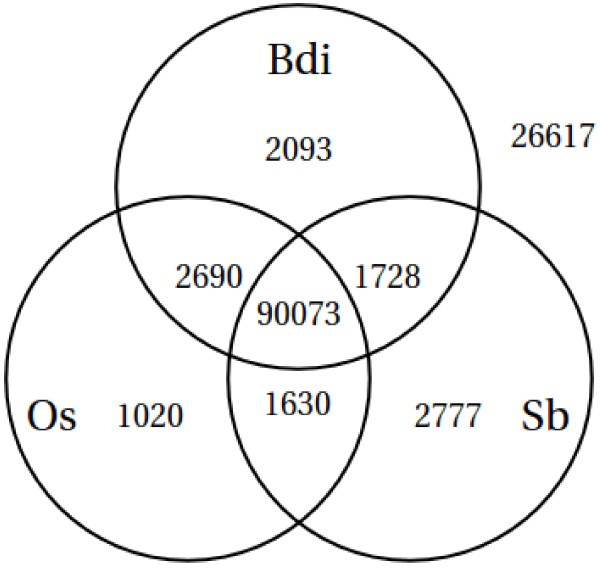
**Assembled transcripts in common with other grasses.** (Bdi: Brachypodium, Os: Oryza sativa, Sb: Sorghum).

The assembled wheat sequences were compared to those available for the published genomes of rice [36], sorghum [37] and brachypodium [38] (see Methods). Just under 80% of the wheat contigs have an apparent homologue in one of these diploids (see Figure 5). In most cases (70%) a homologue was identified in all three species and, quite reasonably given the relative proximity of brachypodium to wheat in the phylogenetic tree, more homologues were identified in brachypodium than in the other two species. We do not believe that all of the 20% of wheat sequences that did not have a match in the other species are unique to wheat; rather, closer inspection reveals that these sequences tend to be those contigs that are shorter than average, and so we think that inadequate sequence length prevented a reasonable match from being identified. This interpretation is supported by the fact that, if only contigs longer than 2000 bases are retained in Figure 5, the proportion of unmatched sequences reduces dramatically to around 1.2%.

In most cases, several wheat sequences are associated with each brachypodium, rice and sorghum sequence in Figure 5 simply because of the polyploid nature of the wheat genome and so this figure does not in itself provide information about the proportion of sequences in brachypodium, rice and sorghum that are represented in wheat. Inspection of the sequence comparison results (see Methods) shows that 88.8% of brachypodium sequences, 89.4% of sorghum sequences and 70.0% of rice sequences are matched by at least one wheat contig. These numbers lend support to the estimates of coverage resulting from the comparison to the FL and TC wheat datasets earlier on.

Finally, the wheat contigs were compared against 23,614 published sequences of full length barley cDNAs [39]. 86,631 wheat sequences (67.4%) were represented in the barley full length cDNAs and 19,762 barley sequences (83.7%) were found to have at least one wheat homologue. One would expect the number of wheat sequences represented in the full length cDNAs to be lower than that for rice, brachypodium and sorghum simply because the barley cDNA dataset [39] does not correspond to the full barley genome. This appears to be the case, however the comparison to barley sequences was performed on the DNA level, while the comparison to the other three genomes was performed on the peptide level, so an accurate quantitative comparison of numbers is not possible.

### Gene Ontology analysis and Pfam domains

The wheat contigs were classified into the gene ontology hierarchy [40] by transferring the GO terms of the best sequence match in rice to each matched wheat sequence, as described in Methods. In this way, 64,071 of the 128,628 wheat contigs inherited 248,403 GO terms. This compares to 18,307 of the 57,624 rice loci annotated with 70,425 GO terms.

The results of this GO analysis for the “Molecular function” ontology are shown in Additional file 3: Table S3. It is noticeable that GO categories associated with binding to RNA (particularly RNA silencing-related proteins DCL3 and HEN1, occurring in the ratios 25/1 and 22/1 in wheat vs. rice), chromatin binding (particularly various regulators of chromosome condensation) and translation factor activity (particularly various translation initiation and elongation factors) appear greatly enriched in wheat compared to rice. It is tempting to speculate that all three categories are enriched in wheat because of its enormous number of transposable elements [41]. This may require an increased need for RNA silencing and/or translational inhibition [42].

The wheat contigs were also scrutinized for occurrences of Pfam domains (see Methods). In total, 65,826 contigs were found to contain 3,073 unique domains. For comparison, the rice coding sequences [36] contained 51,437 sequences with 3,265 unique domains. 3,043 of these domains are in common between the two species. Generally speaking, the number of occurrences of any particular Pfam domain is strongly correlated in wheat and rice (Spearman rank coefficient 0.69; see Additional file 4: Figure S2), indicating that there is no strong evidence of genome wide functional bias associated with the assembly procedure. Some notable exceptions to this are shown in Additional file 5: Table S4, corresponding to off-diagonal points in Additional file 4: Figure S2 with domains associated with transposable elements (e.g. MULE, RVT_2, rve, Plant_trans etc.) being particularly over-represented in wheat, in line with the expectation outlined above [41].

### Homologous cluster analysis

**Figure 6:**
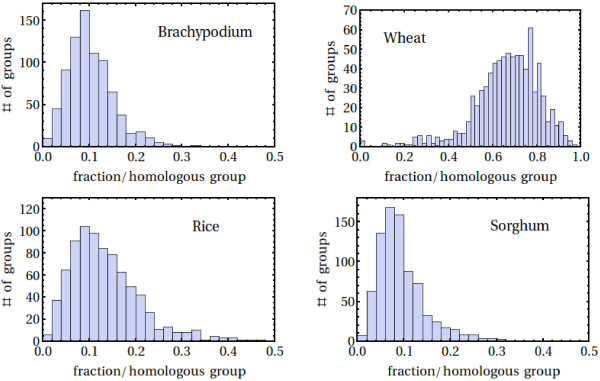
**Sequence content of homologous groups.** Only groups with more than 30 sequences are shown here, with the average content being 10.4% brachypodium, 66.3% wheat, 13.7% rice and 9.6% sorghum.

OrthoMCL [43,44] was used to cluster wheat contigs into 19,086 putative homologous groups together with the peptide sequences of the three sequenced genomes of brachypodium, rice and sorghum, as described in Methods. As shown in Figure 6, the three diploid species contribute, on average, around 11% of the sequences of each group, while wheat contributes the remaining 2/3.

Groups with an abnormally low number of wheat sequences (Additional file 6: Figure S3) include one annotated as subtilisin (45 sequences only present in sorghum) and a group of 44 cytochrome P450s, while groups with an abnormally large number of wheat sequences (Additional file 7: Figure S4) include a group of 55 alanyl-tRNA synthetates and 72 splicing factors, broadly consistent with the over-representation of translation and RNA binding related activities indicated in Additional file 6: Table S3.

## Discussion

Creating a clean assembly of a polyploid transcriptome, where homoeologs have a very high sequence identity, challenges most sequence assembly algorithms. Of the algorithms examined, the overlap-consensus based MIRA algorithm [19-21] was found to give the best results and by a significant margin (see Figure 1). We presume this is due to the multi-pass algorithm implemented in MIRA, which generates a sequence of assemblies, with each iteration learning about possible mis-assemblies from the previous one. Sequence quality estimates, and even platform-dependence of likely sequencing errors, are taken into account by this assembler when making decisions as to which nucleotide changes to interpret as being indicative of a related transcript and which ones to ignore as being likely results of sequencing errors. The whole approach used by MIRA is rather similar, in principle, to the way one might painstakingly construct an assembly by hand.

In this it is rather different to the approach used by short-read assemblers such as Velvet, AbYSS and Trinity. These assemblers are primarily designed to be able to cope with huge read numbers, far exceeding anything that MIRA can deal with. They do this through the use of de-Bruijn graphs constructed from k-mers that are typically shorter than the distance between informative SNPs on the wheat homoeologs. Evidence of multiple homoeologous transcripts is therefore largely determined in a subsequent disentangling of these complex graphs and information about sequence quality is usually ignored in the interests of speed. While this is clearly a valid and highly successful approach, the evidence presented in Figure 1 indicates that it is presently not able to achieve the high precision required to disentangle the wheat homoeologs.

MIRA’s careful approach comes at a high cost in terms of computing memory and time, and made it impractical for us to use the algorithm for a direct assembly of the wheat transcriptome. This was the reason for using the two-step assembly. With ongoing algorithmic improvements to the MIRA assembly algorithm underway, it is hoped that at some stage in the future this somewhat circuitous approach will no longer be necessary. In the meantime, our two-step procedure was sufficiently successful for it to be used for de-novo transcriptome assembly of other complex polyploid organisms, and perhaps for metagenomic samples.

While the two-step approach was developed primarily to deal with the polyploidy of wheat it may have other benefits as well. In particular, as opposed to bacterial or vertebrate genomes that de Bruijn-graph based techniques have been developed for, plant genomes are generally more difficult to assemble because of the greatly increased presence of large gene families. On the other hand it is widely believed that alternate splicing plays less of a role in plants than in animals [45], although this gap is narrowing as knowledge of plant genomics improves [46,47]. One might expect, therefore, that standard short read assemblers developed for microbial/animal genomes may encounter analogous difficulties to the ones discussed here, albeit on a smaller scale, simply because of this increased preponderance of highly similar sequences in plants.

In general, one can estimate that gene duplications predating the evolution of the last common ancestor of the A, B and D genomes of wheat around 5–10 million years ago should exhibit greater sequence divergence than that shown between homoeologs of the same gene, and should therefore be amenable to correct assembly using the parameters that were used here. Conversely, very recent gene duplications (or ancient ones that are under strong selective pressure) may very well confound proper assembly of even diploid plant genomes, regardless of parameters or the assembly algorithm that is used. Generally speaking, no assembly algorithm will be able to distinguish sequences where the average distance between mutations significantly exceeds the effective read length. In this case only increased read length, or construction of libraries with larger insert sizes sequenced as mate-pairs, will lead to increased resolution of related genes. Given that the results presented here indicate that the average distance between SNPs/indels distinguishing wheat homoeologs is around 71 bases, while the effective read length is around 300–350 bases, it is expected that this assembly should typically distinguish gene sequences that arose out of duplication events that are considerably more recent than the polyploidization event characterized by the above time scale.

Having performed the assembly, assessing its characteristics was not straightforward. We were greatly aided by the availability of an EST-based set of validated homoeologous sequence triplets [18,25]. This comparison indicated that differentiation of individual homoeologs in the assembly was excellent, with over 95% of homoeologs represented by an assembled contig. On a cautionary note, however, this validated set is not overly large and there is a danger of possible bias as EST collections tend to be dominated by the most abundantly expressed transcripts. In other words, it is possible that homoeologous sequence triplets with highly asymmetric expression between the homoeologs – and these ones are the hardest to assemble with confidence – are underrepresented in the OM dataset. It may be, therefore, that the true homoeolog specificity of the assembly is somewhat less than the estimation based on the OM sequence set.

Similarly, the comparison to the OM set is only likely to be an approximate indication of the percentage of the total transcriptome covered by the assembly (e.g. there was at least one assembled contig found for every one of the OM sequence triplets). As shown in the Results section, comparison to the FLs and TCs indicates that the true number is more likely to be around 75-80%, dropping somewhat (to perhaps 45-70%) when homoeolog specificity is taken into account. The latter estimate, however, is rather uncertain due to confounding factors, such as residual sequence error as well as variety-specific sequence differences. The lower limit on the number of homoeologous triplets deduced from Figure 3 indicates that the true figure for homoeologous-specific coverage is likely to be towards the upper end of this estimate. It is our expectation that the main reason for the 20-25% reduction from a total coverage of the transcriptome is the fact that not all possible tissues, developmental stages, environmental conditions and stress responses were probed, i.e. not all genes were expressed in our samples. Other factors associated with transcriptome sequencing, such as fluctuations in expression levels insufficiently attenuated through the normalization procedure, or limited number of reads for transcripts expressed at very low levels, may also have had an effect on the final coverage that was achieved.

Broadly speaking, these estimates of coverage are in line with the results obtained when comparing the wheat contigs with available sequences from the rice, sorghum, brachypodium and barley genomes. 70-90% of genes in each of these genomes were found to have a representative in the wheat assembly. Brachypodium, which is the closest relative to wheat among these grasses, has a genome presently estimated to contain just over 31,000 genes. Together, this leads to an estimate that the wheat transcript assembly corresponds to at most ~62,000-80,000 homoeologs (assuming three homoeologs/gene). With a total number of wheat transcripts of 128,628 this in turn implies that our assembly contains 1.6-2.1 transcripts/homoeolog. This estimate is quantitatively consistent both with difference in cumulative sequence length distributions of wheat and rice shown in Figure 2B as well as the relative contribution that the wheat sequences make, on average, to the sequence content of the homologous groups of the grass genomes shown in Figure 6. An assembly with parameters that are less stringent than the ones used here would decrease the number of transcripts per homoeolog, but would also increase the mis-assembly of homoeologs.

Finally, the sequence resource provided here makes it appropriate to carry out similar projects on additional varieties for SNP discovery and genotyping purposes. We are in the process of sequencing the transcriptome of an additional five elite bread wheat lines, results of which will be presented elsewhere. Furthermore, the transcript sequences provided by this work should prove of great use to the annotation of genome-sequencing projects in that they provide valuable information on identifying those parts of the genome that are actually expressed.

## Conclusion

We have used next-generation sequencing combined with an effective parallelization of the sequence assembly process that permits the generation of a high-quality, homoeolog-specific, assembly of the transcriptome of a polyploid species. While the work provides a methodology that can be used for other organisms and/or metagenomic or metatranscriptomic samples, the main outcome is that it makes available, for the first time, a comprehensive compendium of the homoeologous gene sequences of bread wheat. We have deposited these sequences, as well as the raw data used in this study, at NCBI under project number BioProject ID PRJNA76847. The transcript set is also available from the authors on request.

## Methods

### GSFLX Titanium sequencing

The method of Meyer et al. [24] with minor modifications was used for GSFLX Titanium library construction to improve full-length gene coverage by removing coverage bias towards the 3^′^ termini of transcripts.

Briefly, the SMART cDNA synthesis kit (Clontech) was used to produce double-stranded cDNA. Deviations from the manufacturer’s instructions included use of a modified oligo(dT) primer to create first-strand cDNA, and a modified priming strategy during second-strand cDNA synthesis to promote the amplification of larger mRNA molecules. The resulting double-stranded cDNA was normalized using Kamchatka crab duplex-specific nuclease (Evrogen), followed by nebulisation and fragment size-selection.

Next, the size-selected cDNA fragments were blunt-end repaired and a mixture of two partially double-stranded oligonucleotide adapters were ligated to the ends of the cDNA fragments. PCR amplification was performed using different combinations of primers to specifically amplify cDNA fragments corresponding to the 3′-terminal, 5′-terminal and internal regions of mRNA transcripts. The three populations of cDNA fragments were pooled in equimolar amounts and used directly for emulsion PCR to prepare immobilised template for GSFLX Titanium sequencing according to standard Roche protocols. The purpose for amplifying the three different populations of cDNA fragments was to ensure more even sequence coverage along transcripts by reducing coverage bias towards the 3′termini of transcripts caused by the transcription of truncated mRNA during first-strand cDNA synthesis.

### Illumina sequencing

Two types of cDNA library were used for Illumina sequencing. The first library was prepared from the double stranded cDNA used for the Roche GSFLX Titanium sequencing. Briefly, an aliquot of the double stranded cDNA (taken after Kamchatka crab duplex-specific nuclease normalisation and before nebulisation) was used as input for library preparation following the Illumina protocol for preparing samples for sequencing genomic DNA (Part# 1003806 Rev. B, March 2008). The purpose for Illumina sequencing of the same cDNA used for GSFLX Titanium sequencing was to enable correction for known homopolymer errors in pyrosequencing chemistry and to provide high coverage to assist transcriptome assembly. The second library used for Illumina sequencing was prepared from polyA-selected mRNA according to the Illumina protocol for (Part# 15014673 Rev. C, June 2010). Both libraries were prepared from 250–300 bp size selected cDNA fragments and were sequenced on an Illumina GAII_x_ instrument to generate 150-bp paired end reads.

### Quality filtering

Quality trimming of raw GSFLX and Illumina reads was performed using custom Perl scripts according to the following prescription: a) reads were trimmed back if they contained more than three consecutive ambiguous bases, or more than three consecutive bases with a PHRED score <20; b) reads were discarded if the median PHRED score was <20; and c) reads were discarded if the length was <50 bp. Adapters were trimmed off the GSFLX reads using custom scripts and allowing for some mismatches.

### The Ogihara-Mochida (OM) dataset

The quality of the assembly was investigated by comparing our results to the validated homoeolog-specific bread wheat (cv. Chinese Spring) assemblies of Ogihara et al. [25] and Mochida et al. [18]. In that work, 79 genes were identified as being expressed from all three diploid genomes and we selected 65 that could be unambiguously aligned and truncated to completely overlapping sequence triplets for each homoeologous group. In this way, at each informative base a clear decision could be made about whether a particular contig more closely matched the homoeolog on the A, B or D genome. We refer to this set of 65 truncated triplets as the “OM” sequence set. Sequences were compared to the OM set using Blast (E-value <10^-100^, word size 11). Sequences were allocated to individual homoeologs, using %ID as a criterion, by iteratively identifying and removing highest quality matches. For example, if the percent identities P of contigs 1, 2 and 3 with OM sequences A, B and D were P_1A_ = 99, P_1B_ = 98, P_1D_ = 97, P_2A_ = 99, P_2B_ = 100, P_2D_ = 98, P_3A_ = 97, P_3B_ = 97 and P_3D_ = 98, respectively, then first contig 2 would be identified with B, then contig 1 with A and finally contig 3 with D.

### Assembly algorithm performance testing

We filtered reads likely to have originated from the genes in the OM set out of the complete dataset using BlastN (alignment length >100 bases, %ID >95%). The filtered reads were then assembled into contigs using a variety of assemblers: Velvet ([16], Version 1.0.18) and Oases (Version 0.1.18), ABySS ([26], Version 1.2.6) and MIRA ([19,20], Version 3.2.1). Contigs were allocated to OM homoeologs as described above and performance was evaluated using criteria such as %ID to OM, number of matched homoeologs in OM, contig length, and evidence for inter-homoeolog mis-assembly. The latter was assessed by splitting each of the OM homoeologs into three equal pieces and performing the contig-homoeolog matching described above for each piece separately. If the matching was not consistent for all three sequence stretches we took this as evidence of possible chimeric assembly of homoeologs. For each assembly algorithm, a range of assembly parameters was investigated. In the case of the de-Bruijn graph-based assemblers this consisted of a range of k-mer lengths (25, 29, 35, 39, 45, 49, 55 and 99 bases), while for MIRA we performed assemblies with parameters as listed in Additional file 1: Table S1. Finally, we also compared results to those obtained with Trinity ([27], Version 5-19-2011), a transcriptome assembler that has become available more recently.

### Assembly pipeline

We adopted a two-phase approach to the assembly by first using a largely homoeolog-insensitive assembly to group reads into clusters so that a subsequent time- and memory- intensive homoeolog-sensitive assembly could be parallelized. Of particular interest for the first-pass assembly was the maximization of total sequence coverage rather than homoeolog specificity, as the former could not be improved upon in any subsequent homoeolog-specific read assembly. For this reason we chose Velvet/Oases for the initial assembly (see Figure 1B). Sequence coverage, gauged by comparing the total length of all alignments to the total length of all sequences in the OM set, was maximized by choosing a k-mer size of 39 (see Additional file 8: Figure S1).

This Velvet/Oases assembly was used to partition the complete set of reads into clusters. First, the assembled contigs were themselves transitively clustered if they shared a common subsequence of at least 32 bases. This clustering was carried out to mitigate the effects of unwanted separation into homoeologs during this first round. Next, all reads were assigned to these clusters using the same criterion. In total, 12,485,611 reads (i.e. 78% of the total) were grouped into 34,990 clusters. In a limited number of cases, this grouping was not unique as reads had separate 32-mers in common with more than one cluster. In these cases, the read was assigned to more than one cluster. The Velvet/Oases transcripts giving rise to the clusters in the first place were not used in any subsequent analysis.

Next, the reads were assembled using MIRA. Extensive investigation of parameter settings (see Figure 1) using the subset of reads relevant to the OM set and quality assessment described in the main text indicated that standard parameter settings were sufficient for optimum homoeolog-specific assembly, with the exception that the minimum relative score for assembly of two reads (AL:mrs) was increased significantly from default settings to 97%. For the Illumina paired-end reads, we allowed for an insert size between 1 and 500 bases.

Assembly of the smaller clusters as well as the 10 largest clusters was performed sequentially on a 64-bit, 20 GB RAM, 30 GB Swap, 2.3 GHz Quad-core AMD Opteron processor workstation. Assembly of the rest of the 10,000 largest clusters was done in parallel on the *Australian Research Collaboration Service*’s compute cloud [48]. Job submission was carried out through custom-written Python scripts, and the cloud had available to it 1150 CPUs that were configured to run with either 1 or 4 GB RAM each. Most clusters were assembled in the order of a few minutes and the total execution time would have been around 16 days had the assemblies been carried out sequentially.

### Annotation

Both BlastX and BlastN was used to obtain functional annotation by comparing against rice cDNAs (PlantGDB [49], Release 6.1; BlastN E-value < 10^-10^, BlastX E-value < 10^-3^). Map locations from the NSF EST deletion mapping project [50] were added when available. In total, almost 78.5% sequences received annotation in this way. Assembled sequences, as well as the original raw reads, were deposited at NCBI under project number BioProject PRJNA76847 after performing additional sequence trimming of a small number of contigs that showed evidence of insufficient Illumina adapter trimming. Annotated assembled contigs may also be obtained from the authors. As a naming convention for the contigs we adopted KukriC*x*_*y*, where *x* indicates the Velvet/Oases cluster while *y* indicates the *y*’th contig within that cluster.

### Comparison to Harvard TCs as well as full length cDNAs

Assemblies were compared to a collection of 93,508 wheat tentative contigs (TCs) of the *Gene Index Databases*[31,32] using BlastN ([51]; E-value <10^-50^, wordsize 20, with varying lower cutoffs for %ID of the alignment was used). The TCs were assembled by the DFCI group from a little more than 1 million wheat ESTs, using the cap3/cap4/Paracel assembly algorithm by demanding an overlap of at least 40 bases with 94 %ID [32]. These parameters would have ensured that in most cases homoeologous ESTs end up in the same cluster. While we are not aware of any systematic study of the performance of the subsequent Paracel assembly algorithm when used for a polyploid genome, its base-calling procedure [52,53] suggests that there is a clear danger for homoeologs to be missed when expression levels between the three copies is asymmetric. (We note in passing that Paracel does not produce ambiguous consensus sequences, as is evidenced by the fact that none of the ca. 8.19 × 10^7^ nucleotides in the wheat TCs is represented by an ambiguous IUPAC nucleotide symbol). In addition, the allelic diversity of the ESTs used in the TCs may also have been a complicating factor.

Comparison to the TriFLDB full length cDNA clones [29,30] was performed in the same way.

### Homoeolog specificity

The comparison to data from genomic wheat sequencing projects was performed by first using the Blast server at CerealsDB.uk.net in order to extract reads of relevance to 19 of our contig clusters. These reads were mapped to sets of 3 mutually overlapping contigs using the Geneious software package. After careful manual editing, required to remove obvious introns as well as large numbers of clear homopolymer-associated sequencing errors in individual genomic 454 reads, we scored the informative positions of the Kukri contig triplets against the reads. If reads could be clearly associated over more than 2 consecutive informative positions with one Kukri contig, but to another Kukri contig in an adjacent part of the read, we interpreted this as evidence of chimeric assembly.

### Comparison to other grass genomes

Peptide sequences for rice were obtained from ([28], Version 6.1; 67,393 sequences), for brachypodium from ([54], Version 1.2; 31,029 sequences) and for sorghum from ([55], Version 79; 29,448 sequences). Sequence comparisons were performed using NCBI BlastX (E-value ≤ 10^-3^, alignment length ≥ 20). Barley full-length cDNA sequences were downloaded from NCBI ([3]; author Matsumoto, organism barley; 23,614 sequences) and compared using NCBI BlastN (E-value ≤ 10^-10^, alignment length ≥ 60).

### Gene ontology analysis and Pfam domains

GO assignments for the rice peptides were downloaded from [28] and processed so that, for each peptide, only the most specific assignments were kept. In this way, we avoided double-counting associated with redundant assignments of peptides in the GO hierarchy. The wheat contigs were compared with the peptide sequences using NCBI BlastX (E-value ≤ 10^-3^, alignment length ≥ 20). The best match for each contig was then used to transfer the GO annotation from rice to wheat.

Rice Pfam protein domains were downloaded from the Pfam database [56]. Both the wheat contigs as well as rice coding sequences were compared to these domains using NCBI BlastX (E-value ≤ 10^-3^). In order to avoid redundant hits, for each query-target pair only the Blast hit with the lowest E-value was retained, i.e. multiple identical Pfam domains within a contig were only counted once.

### Homologous cluster analysis

OrthoMCL [43,45] was used to cluster the rice, sorghum and brachypodium peptide sequences described earlier into 23,928 homologous clusters (clustering parameters: E-value ≤ 10^-5^, cluster inflation value I = 5). Results for the cluster size distribution were found to be relatively stable with respect to changes in these parameters. Wheat nucleotide sequences were assigned to each of these clusters through the use of best-hit BlastX matching (E-value ≤ 10^-5^, HSP alignment length ≥ 20). When the OrthoMCL clusters of the best-hit to rice, sorghum and brachypodium were not identical, which occurred in a relatively small number of cases, these clusters were merged. All sequences in any of the four species annotated as “transposon”-related were eliminated, resulting in a homologous clustering with 19,086 transcript groups.

## Competing interests

The authors declare that they have no competing interests.

## Authors’ contributions

AWS performed assemblies, quality checks and drafted the manuscript. MJH, KF and SK performed sequencing runs and assisted in drafting the manuscript. PL was involved in conceiving the project. UB performed assemblies on the compute cloud and assisted in drafting the manuscript. All authors read and approved the final manuscript.

## Supplementary Material

Additional file 1 **Table S1.** Is a table containing parameters used for optimizing the MIRA assemblies shown in Figure 1. Click here for file

Additional file 2 **Table S2. **Is a table showing the comparison of assembled wheat sequences to the OM data set. Click here for file

Additional file 3 **Table S3. **Is a table containing statistics of the Gene Ontology characterisation of the. Click here for file

Additional file 4 **Figure S2. **Contains a figure comparing the number of Pfam domains found in wheat as. Click here for file

Additional file 5 ** Table S4.** Contains similar statistics pertaining to the occurrence of Pfam domains. Click here for file

Additional file 6 ** Figure S3. **Is a figure showing the contribution of under-represented wheat sequences to homologous clusters of the four grass species considered here. Click here for file

Additional file 7 ** Figure S4.** Is a figure showing the contribution of over-represented wheat sequences to homologous clusters of the four grass species considered here. Click here for file

Additional file 8 **Figure S1.** Is a figure showing the average alignment length and %ID of the Velvet/Oases assemblies compared to the OM sequence set. Click here for file
