# Supplementary material for: Transcriptome-scale homoeolog-specific transcript assemblies of bread wheat
Source: BMC Genomics. 2012 Sep 19;13:492. doi: 10.1186/1471-2164-13-492 (PMC3505470; doi:10.1186/1471-2164-13-492)
Supplement: Additional file 1 — Table S1. Is a table containing parameters used for optimizing the MIRA assemblies shown in Figure 1. [file 1471-2164-13-492-S1.docx]

**Supplementary Table 1**: Parameters used with the MIRA assembly algorithm

| **Assembly** | **Parameters** |
| --- | --- |
| M_6_ | default parameters |
| M_7_ | default parameters, except: SOLEXA AL:mrs=95 ED:ace=yes |
| M_8_ | default parameters, except: SOLEXA AL:mrs=95:mo=60 ED:ace=yes |
| M_9_ | default parameters, except: AS:rbl=8 |
| M_10_ | default parameters, except: AS:nop=8 |
| M_11_ | default parameters, except: SOLEXA ED:ace=yes |
| M_12_ | default parameters, except: SOLEXA AL:mrs=95 |
| M_13_ | default parameters, except: SOLEXA AL:mrs=99 |
| M_14_ | default parameters, except: SOLEXA AL:mrs=70 |
| M_15_ | default parameters, except: CO:mrpg=3 |
| M_16_ | default parameters, except: CO:mnq=30 |
| M_17_ | default parameters, except: SOLEXA CO:mgqrt=35 454 CO:mgqrt=30 |
| M_18_ | default parameters, except: SOLEXA AL:mrs=95, no 454 reads |
| M_19_ | default parameters, except: SOLEXA AL:mrs=97 |
| M_20_ | default parameters, except: SOLEXA AL:mrs=98 |
