# Supplementary material for: Transcriptome-scale homoeolog-specific transcript assemblies of bread wheat
Source: BMC Genomics. 2012 Sep 19;13:492. doi: 10.1186/1471-2164-13-492 (PMC3505470; doi:10.1186/1471-2164-13-492)

**Supplementary Figure 2.** Comparison of number of occurances of Pfam domains in rice and wheat. The abundance of domains that are absent in one of the two species has been regularized to 10^-1^ in order to be able to show them on this logarithmic plot. A general strong correlation between the number of Pfam domains in rice and wheat is clearly visible.


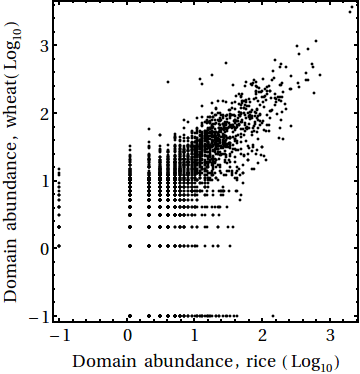

Supplement: Additional file 4 — Figure S2. Contains a figure comparing the number of Pfam domains found in wheat as. [file 1471-2164-13-492-S4.docx]
