# Supplementary material for: Transcriptome-scale homoeolog-specific transcript assemblies of bread wheat
Source: BMC Genomics. 2012 Sep 19;13:492. doi: 10.1186/1471-2164-13-492 (PMC3505470; doi:10.1186/1471-2164-13-492)

**Supplementary Figure 4.** Homologous clusters with the highest proportion of wheat sequences. Clusters are arranged with decreasing numbers of total sequences, with a minimum cluster size of 30 sequences. Annotation of the bulk of the sequences in the clusters is shown in the legends.


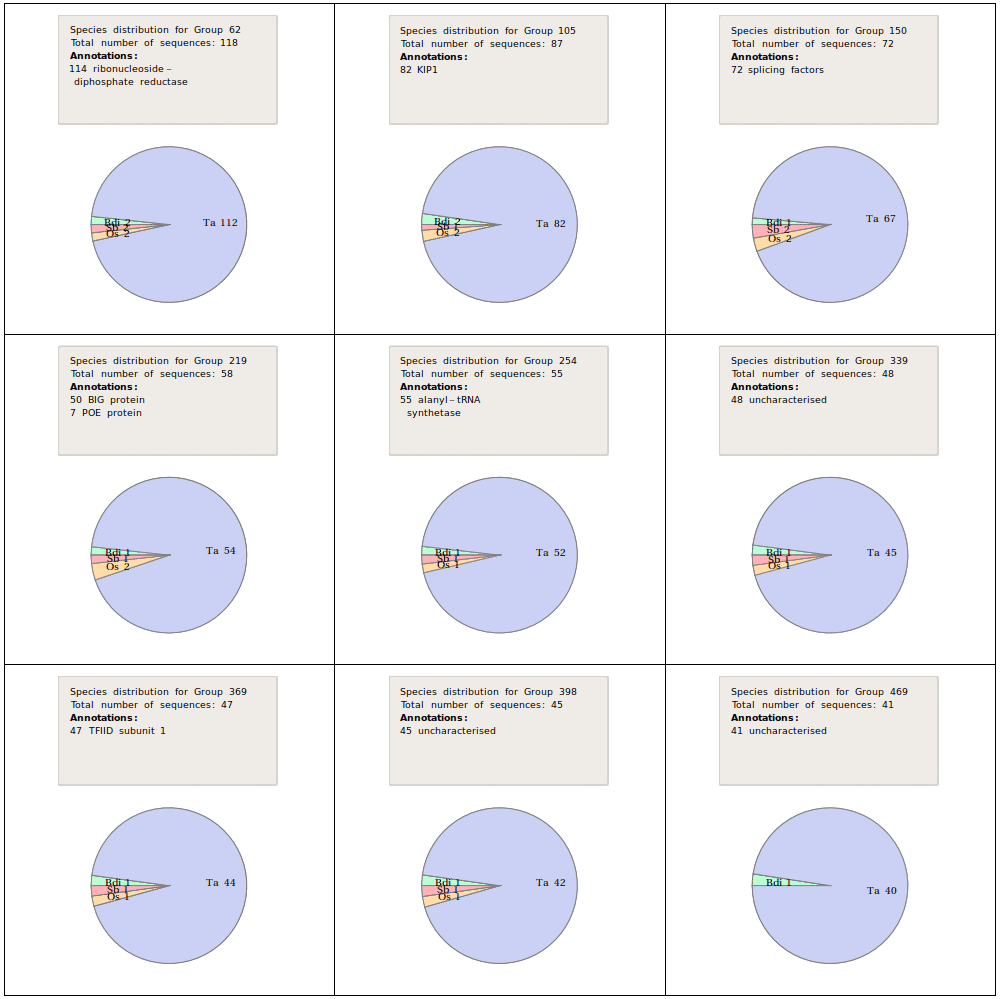

Supplement: Additional file 7 — Figure S4. Is a figure showing the contribution of over-represented wheat sequences to homologous clusters of the four grass species considered here. [file 1471-2164-13-492-S7.docx]
