# Supplementary material for: Transcriptome-scale homoeolog-specific transcript assemblies of bread wheat
Source: BMC Genomics. 2012 Sep 19;13:492. doi: 10.1186/1471-2164-13-492 (PMC3505470; doi:10.1186/1471-2164-13-492)

**Supplementary Figure 1.** Comparison of Velvet/Oases assemblies to the OM set. The total length of alignments (left, as fraction of total sequence) to the OM set as well as average %-Identity of Blast matches (right) as a function of k-mer length is shown. With increasing k-mer size the assemblies become more homoeolog-specific, at the expense of total sequence coverage.


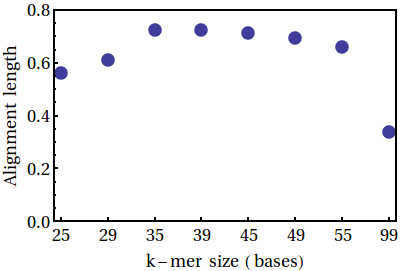

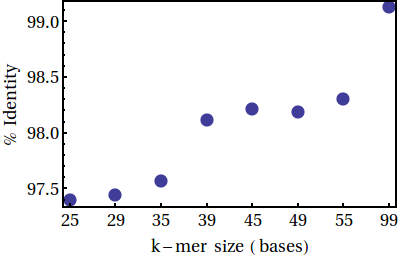

Supplement: Additional file 8 — Figure S1. Is a figure showing the average alignment length and %ID of the Velvet/Oases assemblies compared to the OM sequence set. [file 1471-2164-13-492-S8.docx]
